# No Evidence for Light-Induced Embolism Repair in Cut Stems of Drought-Resistant Mediterranean Species under Soaking

**DOI:** 10.3390/plants11030307

**Published:** 2022-01-24

**Authors:** Martina Tomasella, Sara Natale, Francesco Petruzzellis, Sara Di Bert, Lorenzo D’Amico, Giuliana Tromba, Andrea Nardini

**Affiliations:** 1 Dipartimento di Scienze della Vita, Università di Trieste, Via L. Giorgieri 10, 34127 Trieste, Italy; martina.tomasella@units.it (M.T.); sara.natale@phd.units.it (S.N.); fpetruzzellis@units.it (F.P.); sara.dibert@studenti.units.it (S.D.B.); 2 Dipartimento di Scienze Agroalimentari, Ambientali e Animali, Università di Udine, Via delle Scienze 91, 33100 Udine, Italy; 3 Elettra-Sincrotrone Trieste, Area Science Park, 34149 Basovizza, Italygiuliana.tromba@elettra.eu (G.T.); 4 Dipartimento di Fisica, Università di Trieste, Via A. Valerio 2, 34127 Trieste, Italy

**Keywords:** stem photosynthesis, hydraulic recovery, soaking, X-ray micro-CT, bark water uptake, embolism

## Abstract

(1) Recent studies suggested that stem photosynthesis could favor bark water uptake and embolism recovery when stem segments are soaked in water under light conditions, but evidence for this phenomenon in drought-resistant Mediterranean species with photosynthetic stems is missing. (2) Embolism recovery upon immersion in water for 2 h–4 h under light was assessed (i) via a classical hydraulic method in leafless *Fraxinus ornus* and *Olea europaea* branch segments stressed to xylem water potentials (Ψ_xyl_) inducing ca. 50% loss of hydraulic conductivity (PLC) and (ii) via X-ray micro-CT imaging of the stem segments of drought-stressed potted *F. ornus* saplings. Hydraulic recovery was also assessed in vivo in intact drought-stressed *F. ornus* saplings upon soil re-irrigation. (3) Intact *F. ornus* plants recovered hydraulic function through root water uptake. Conversely, the soaked stem segments of both species did not refill embolized conduits, although Ψ_xyl_ recovered to pre-stress levels (between −0.5 MPa and −0.2 MPa). (4) We hypothesize that xylem embolism recovery through bark water uptake, even in light conditions, may not be a common phenomenon in woody plants and/or that wounds caused by cutting short stem segments might inhibit the refilling process upon soaking.

## 1. Introduction

Most terrestrial plants rely on root-level water absorption to maintain their hydration status. However, water can also be absorbed from the surface of aboveground plant organs under particular biophysical conditions, i.e., when liquid water is wetting plant surfaces or the water potential of the surrounding atmospheric boundary layer is higher than that of cells [1]. This occurs when water vapor pressure in the air is at (or close to) saturation and, most importantly, when liquid water forms or falls on a plant surface due to fog, rain or snow melting, allowing local rehydration and partial xylem tension relief, especially when plants are experiencing a soil water deficit [2].

Water uptake through leaves has been widely observed in many plant lineages [3,4]. Leaf trichomes, depending on their density, composition and structure, are the major media for water absorption in a Mediterranean (*Quercus ilex*) and a temperate (*Fagus sylvatica*) species [5,6]. In addition, in two species without leaf trichomes or hydathodes, open stomata have been observed to play a major role in water uptake over cuticles when exposed to fog [7]. Leaf water absorption can also be involved in the recovery of leaf [8] and stem [9,10] hydraulic functions after drought- or frost-induced xylem embolism.

The stems of woody plants, even when covered with suberized tissue, are also able to absorb water when the bark becomes wet, allowing partial xylem tension relief [11]. There is some evidence that leafless dehydrated branches soaked in water for at least some hours partially recover their xylem hydraulic function in a conifer (*Sequoia sempervirens*, [12]) and in an angiosperm (*Salix matsudana*, [13]). Xylem hydraulic recovery would require radial water movement from the phloem to the xylem through parenchyma rays, as proven in a dying experiment on wetted stems [11]. Liu et al. [13] soaked short *S. matsudana* stem segments in water and observed faster embolism repair (within 2 h) when under light conditions compared to dark conditions, where partial refilling nevertheless occurred. The enhanced refilling effect under light was ascribed to the sugars produced by stem photosynthesis, which provide the driving (osmotic) force for the process. In fact, embolism repair can occur as the result of a water potential gradient between the xylem apoplast, where sugars are accumulated, and parenchyma cells surrounding the conduits [14,15,16]. Independently of the mechanisms involved in the process, such soaking experiments on short leafless stem segments on other woody species performing stem photosynthesis are underrepresented, and they might constitute an interesting experimental model to investigate the biology of post-drought hydraulic recovery.

In the past decade, some methodological issues have arisen regarding stem hydraulic vulnerability assessment and the study of embolism recovery via classic hydraulic methods due to the possible overestimation of embolism rates, which may be generated when cutting xylem under tension [17]. At the same time, prolonged xylem relaxation prior to hydraulic measurements, which is suggested to avoid artefactual embolism appearance, can favor refilling, leading to the underestimation of embolism levels [18]. In well-established sample preparation procedures for hydraulic measurements, stems are often kept under water prior to hydraulic measurements, even for long time intervals, based on the principle that the very low pressure head upon immersion would avoid embolism dissolution [19]. However, the above-mentioned soaking experiments in short leafless branch segments [12,13] indicated that the active accumulation of solutes at the wood parenchyma–conduit interface drives water into the apoplast and might refill the conduits under soaking. If this happens, soaking stems for a prolonged period would induce overestimation of xylem hydraulic conductivity.

These controversies can be overcome using X-ray micro-computed tomography (micro-CT), an important tool to visualize embolized xylem conduits at a proper resolution and to quantify in vivo xylem vulnerability, as well as possible hydraulic recovery in intact plants [20,21]. Therefore, this method has been exploited to validate destructive hydraulic methods (e.g., [22,23]) and related sample preparation procedures [24].

In this study, we hypothesized that bark water uptake in the presence of light would induce the recovery of hydraulic function in two embolism-resistant Mediterranean angiosperm species performing stem photosynthesis, namely, *Fraxinus ornus* and *Olea europaea*. We tested this hypothesis both with a classical hydraulic method in dehydrated leafless cut branches of adult plants of both species, and in vivo through X-ray micro-CT imaging of segments of drought-stressed potted young saplings of *F. ornus*. There is evidence suggesting that *O. europaea* is capable of partial hydraulic recovery following drought-induced xylem embolism [18], making this species a good candidate to investigate the eventual process of conduit refilling under soaking conditions. Similarly, intact *F. ornus* plants have already been reported to recover hydraulic function when the soil is re-watered after substantial drought-induced loss of xylem hydraulic conductivity [25], but in vivo evidence is missing. For this reason, we additionally tested the hydraulic recovery capability of this species through root water uptake via micro-CT imaging of intact potted plants.

## 2. Results

**Figure 1:**
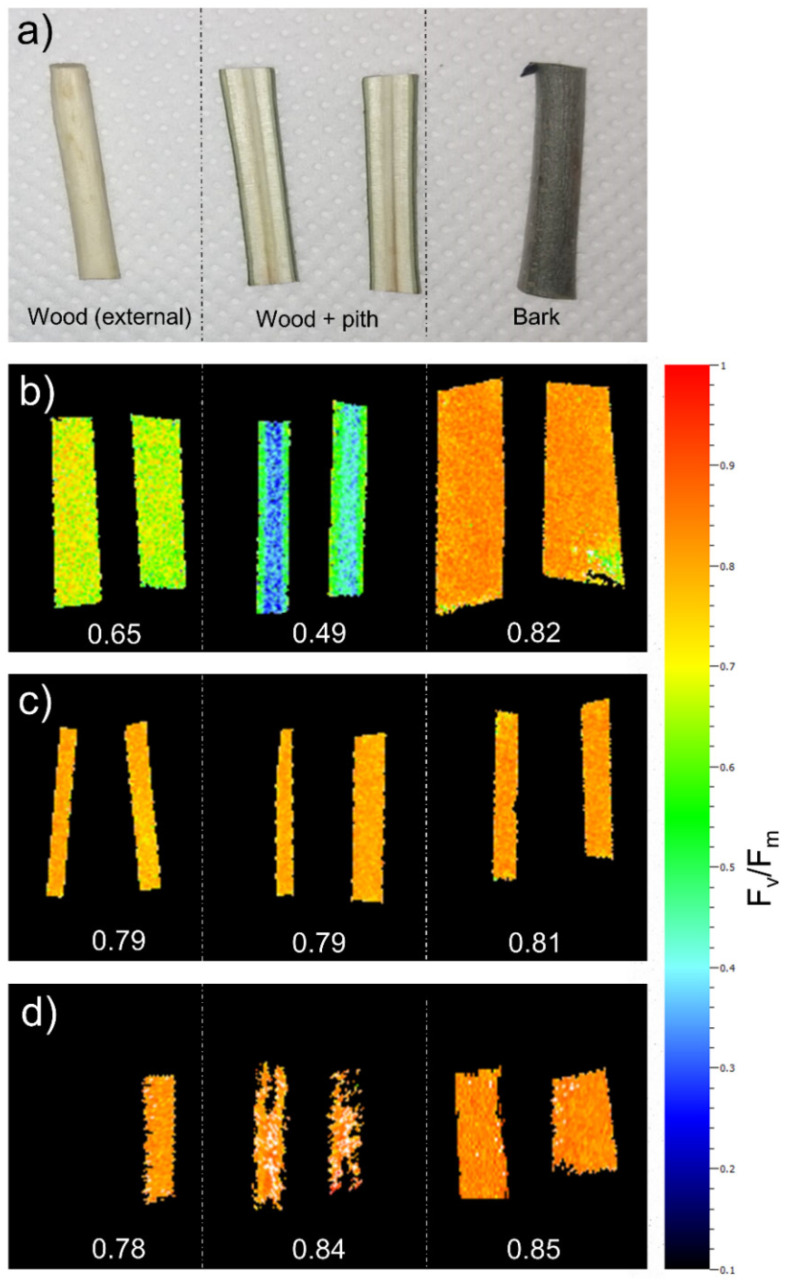
Maximum quantum yield of PSII (F_v_/F_m_) measured in branch/stems used for the experiment. Wood (exposing the side next to the vascular cambium), wood + pith (exposing the cut side) and bark were analyzed separately (**a**). Measurements were performed on 2-year old *F. ornus* branch segments (**b**), in 1-year old *O. europaea* branch segments (**c**) and in 1-year old stems of *F. ornus* saplings (**d**). Values in b–c are the average value of the sample.

The stem segments used for the soaking experiments, i.e., 2-year-old *F. ornus* and 1-year-old *O. europaea* branch portions, as well as the 1-year-old stem portions of *F. ornus* saplings, showed a relatively high capability of performing photosynthesis (Figure 1). In particular, the maximum quantum yield of PSII (F_v_/F_m_) in the outer bark was about 0.8 in all three different samples. Similar values were also measured in the outer wood and in the longitudinal section of the wood (sapwood + pith) of the 1-year-old branch and stem segments of both species. Lower (but still relatively high) F_v_/F_m_ values were measured in the outer wood (0.65) and in the longitudinal section of the wood (0.49) of *F. ornus* 2-year-old branch portions.

### 2.1. Hydraulic Measurements

**Figure 2:**
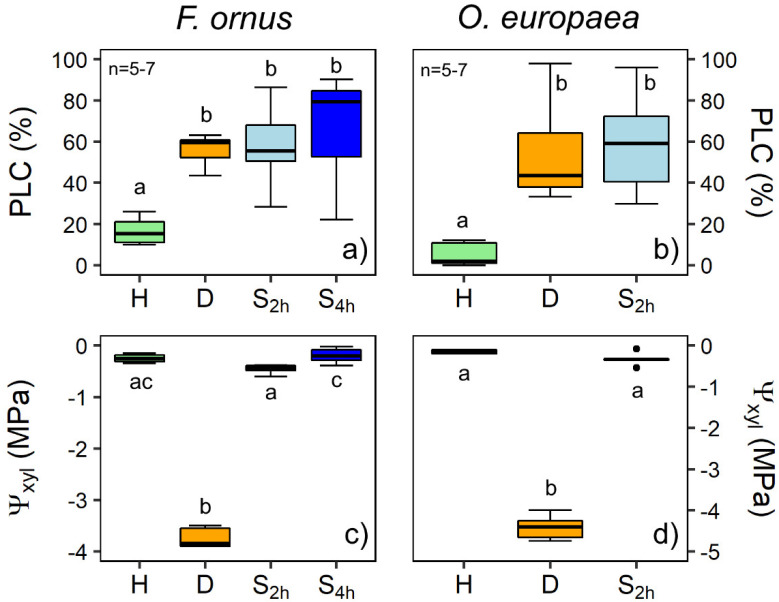
Percentage loss of xylem hydraulic conductivity (PLC, (**a**,**b**)) and xylem water potential (Ψ_xyl_, (**c**,**d**)) measured in *F. ornus* (**a**,**c**) and *O. europaea* (**b**,**d**) in hydrated (H, *n* = 5), drought-stressed (D, *n* = 5–6) and soaked (for 2 and 4 h, S_2h_ and S_4h_, respectively, *n* = 6–7) stems. Note the different scales for Ψ_xyl_ between the two species. The 4 h soaking treatment was performed only for *F. ornus*. Different letters denote statistically significant differences among groups (*p* < 0.05).

Some native embolism was detected in the hydrated branches of *F. ornus* and *O. europaea* (H samples), with PLC averaging 17% and 5%, respectively (Figure 2). After dehydration on the bench, PLC at the target Ψ_xyl_ (−3.7 MPa and −4.4 MPa in *F. ornus* and *O. europaea*, respectively) significantly increased to 56 ± 4% in *F. ornus* and to 54 ± 10% in *O. europaea*. After the soaking treatment in distilled water for 2 h (S_2h_ samples), Ψ_xyl_ increased to pre-stress levels, averaging −0.46 MPa in *F. ornus* and −0.33 MPa in *O. europaea*, but stem PLC did not recover in either species. Moreover, prolonged immersion in water (four hours, S_4h_ stems) did not induce xylem hydraulic recovery in *F. ornus,* albeit xylem tension further and significantly decreased with respect to the S_2h_ samples (Ψ_xyl_ = −0.19 ± 0.06 MPa, *p* < 0.05).

### 2.2. Micro-CT Analyses

**Figure 3:**
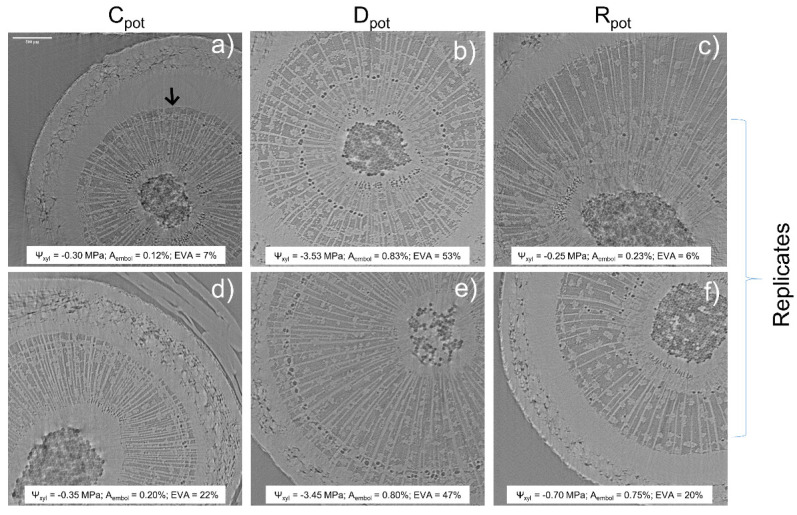
Transverse reconstructed images of the 1-year-old stem portion obtained with X-ray micro-CT in well-hydrated (C_pot_, (**a**,**d**)), drought-stressed (D_pot_, (**b**,**e**)) and re-irrigated (R_pot_, (**c**,**f**)) intact *F. ornus* plants. Ψ_xyl_ = xylem water potential, A_embol_ = percentage of embolized sapwood area, EVA = percentage of embolized vessel area; all calculated excluding the immature sapwood close to the cambium (see arrow delimiting mature/immature sapwood in (**a**)).

The micro-CT experiment on the potted plants of *F. ornus* proved the capability of xylem hydraulic recovery in intact plants through root water uptake 24 h after soil rehydration to field capacity. In fact, the embolized vessel area (EVA) was around 40–50% in drought-stressed (D_pot_) plants, which reached Ψ_xyl_ of −3.50 MPa, and it was restored to pre-stress control (C_pot_) values in the two re-irrigated (R_pot_) plants (6% and 21% in the two scanned plants, Figure 3), which relieved Ψ_xyl_ to −0.25 MPa and −0.70 MPa.

The consecutive micro-CT scans performed on the 2-year-old *F. ornus* drought-stressed (D) plants after each progressive sample preparation step are shown in Figure S1. The first cut at the base of the stem did not significantly increase the embolized sapwood area (A_embol_) or EVA. However, the second cut made to obtain the final D segment significantly increased EVA from 44 ± 7% (measured in the shoots cut at the base) to 63 ± 11%, with an overall increase of 27% compared to the intact plant (Figure S2).

**Figure 4:**
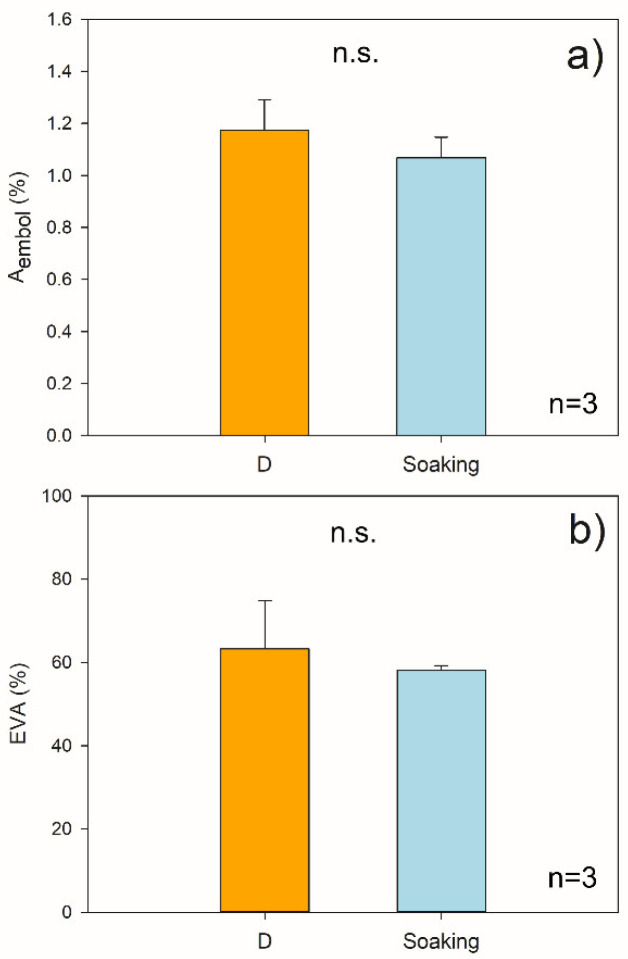
Percentage of embolized sapwood area (A_embol_, (**a**)) and percentage of embolized vessel area (EVA, (**b**)) measured in micro-CT transverse reconstructed images of *F. ornus* stem segments. D = segments of drought-stressed plants (*n* = 3). Soaking = segments of additional D plants scanned upon soaking in water for 2 h (*n* = 3). Values are means ± SE. n.s. = difference not significant among groups.

The stem segments did not refill their embolized conduits after the 2 h soaking treatment under light, as the values of A_embol_ and EVA (1.07 ± 0.06% and 58 ± 1%, respectively) were similar to those calculated for D stem segments (*p* > 0.05; Figure 4 and Figure S3).

## 3. Discussion

### No Evidence for Hydraulic Recovery in Stem Segments upon Soaking

The in vivo micro-CT analyses showed that *F. ornus* plants can recover xylem hydraulic function through root water uptake after experiencing substantial embolism levels under drought, confirming the previous classical hydraulic measurements of cut stems [25]. In addition, embolism recovery occurred even though plants were still experiencing overall negative xylem pressure, supporting previous observations. However, the leafless stem segments of *F. ornus* and *O. europaea* that reached about 50% PLC (i.e., the same target PLC used in the pot re-irrigation experiment) did not recover xylem hydraulics after immersion in water under light conditions (Figure 2). This outcome was additionally validated by the micro-CT scans of the *F. ornus* cut stems, obtained from the drought-stressed potted saplings (Figure 4 and Figure S2).

The hydraulic measurements of *F. ornus* branches (Figure 2) were in agreement with the micro-CT measurements of intact plants (Figure 3), confirming the lack of cutting artifacts. Likewise, the PLC values obtained in our study for *O. europaea* at the target Ψ_xyl_ were consistent with the vulnerability curves obtained for the same species both via hydraulic measurements and X-ray micro-CT scans [26,27], making us confident that sample preparation artefacts were avoided. However, with the methodological test performed at the micro-CT beamline on the dehydrated saplings of *F. ornus*, we aimed to examine if the sample preparation procedure used to obtain the final stem segments for soaking would induce additional non-native embolism. The progressive cuts made on the stem of the potted saplings increased the embolized vessel area (Figure S1 and Figure S2). In this test, we first made a cut underwater at the base of the shoot, and then a second cut was made at a distal position (ca. 20 cm from the previous one) to obtain a stem segment in the basal part of the stem. Given that the maximum vessel length of the saplings (40 cm on average) was higher than the obtained stem segment, it is likely that air entry upon cutting under substantial xylem tension induced embolism in the stem portion where the micro-CT scans were made (at 8 cm–9 cm from the first cut). Given that we aimed to perform the soaking experiment on the lower 1-year-old stem portion and that the plants were relatively small, we were forced to obtain the stem segment in that proximal stem region. With this simple test, we underline that experiments with in vivo imaging techniques should be further exploited to better identify the possible artefacts of sample preparation. However, the results of our cutting test do not influence the outcome of the soaking experiment because the samples scanned prior (D) or after (Soaking) immersion in water (Figure 4) were prepared with the same procedure.

The results of the soaking experiments contradict those obtained for *S. matsudana* stem segments soaked in water under light conditions, where bark water uptake contributed to refill about 75% of the previously embolized vessels after 2 h of immersion [13]. Here, we also showed that *F. ornus* and *O. europaea* stems can perform photosynthesis both at wood and bark levels (Figure 1), which should provide additional sugars to enhance the process of water uptake by generating an osmotic gradient.

There is evidence that local xylem tension relief is important to refill xylem conduits [28,29]. In *S. sempervirens*, partial hydraulic recovery was observed upon soaking when branch water potential slightly increased with respect to dehydrated conditions, but it was still quite negative (ca. −4 MPa, [12]). Similarly, recovery under substantial tension occurred in *Picea glauca* leafy twigs exposed to fog [10], suggesting possible localized hydraulic isolation in the xylem. Xylem tension in our two study species was even relaxed to Ψ_xyl_ above −0.5 MPa (Figure 2). These values are comparable to those measured in the intact potted plants of *F. ornus*, which recovered xylem hydraulics when re-irrigated in pots (Figure 3). Therefore, we note that the residual xylem tension of the cut stems was comparable to that of intact plants, and it was expected to promote the refilling of conduits through bark water uptake.

Earles et al. [12] suggested that tall trees, such as redwoods, which, due to their height, must develop low Ψ_xyl_ even under well-hydrated conditions, may profit from bark water uptake during prolonged fog periods to partially restore xylem functionality. It is suggested that trees adapted to climates with prolonged periods of rain and/or fog [30] and trees at the timberline with crowns covered with snow that melts in spring [9,31] may have evolved bark characteristics that favor water uptake and subsequent hydraulic recovery. Indeed, radial water uptake may depend on outer (rhytidome) and/or inner (phloem) bark characteristics. For example, bark porosity and density influence hygroscopicity [32], while the hydrophobicity of cork cells in the outer bark limits water absorption [33,34]. It is possible that species adapted to Mediterranean climates, commonly not exposed to prolonged fogs/rainy periods, may not have evolved such bark characteristics that would favor radial water uptake. However, we might rule out this hypothesis because, although we did not directly measure bark water uptake, the marked Ψ_xyl_ relief upon soaking indicated substantial stem rehydration (Figure 2b). Alternatively, sample preparation/experimental conditions could have been responsible for the lack of hydraulic recovery. Previous experiments have highlighted the role of phloem in xylem hydraulic recovery [35]. In particular, stem girdling in *O. europaea* prevented embolism refilling upon branch rehydration, while hydraulic recovery was observed in samples with intact phloem [18]. Hence, it is possible that the wounds caused by the preparation of stem segments for soaking had similar inhibitory effects on the refilling process. Tests on different species could help improve our understanding as to whether refilling through bark water uptake might be species specific, as well as the possible limits of the soaking method when applied to cut stem segments.

## 4. Materials and Methods

### 4.1. Plant Material

The experiment was carried out on two Mediterranean tree species, namely, manna ash (*Fraxinus ornus* L.) and olive (*Olea europaea* L.), between mid-June and the end of July 2021. In order to test the hydraulic recovery capability upon soaking under light conditions, embolism was measured both with a classical hydraulic conductivity apparatus and in vivo via micro-CT, but on different plant material. Hydraulic measurements were performed on sun-exposed branches taken from several *F. ornus* trees and one *O. europaea* tree growing in the Botanical Garden of the University of Trieste (Italy, 45°39′40.9″ N, 13°47′40.1″ E). For micro-CT experiments, 2-year-old and 1-year-old *F. ornus* saplings provided by a local public nursery (Vivai Pascul, Regional Forestry Service, Tarcento, Italy) were transplanted in 3.4 L and 1 L pots in March 2020 and 2021, respectively. Pots were filled with a lightweight substrate for green roof installations (for the 2-year-old plants) or with red soil (for the 2-year-old plants) sampled from a vineyard in the Italian Karst (Duino-Aurisina, Italy). Plants were grown in a greenhouse of the University of Trieste, supplied with tap water twice a day to soil field capacity through a clock irrigation system.

To check the capability of the study species to perform bark and wood photosynthesis, some stem segments were analyzed with an imaging PAM chlorophyll fluorometer (Photon Systems Instruments, Brno, Czech Republic) to obtain the maximum quantum yield of PSII (Fv/Fm). Stem segments of approx. 2 cm length were dark adapted for 1 h, longitudinally sectioned and positioned on a plate with a layer of paper towels, keeping them hydrated through partial immersion in a film of water. Outer bark, outer xylem and xylem longitudinal section (sapwood + pith) were measured (see Figure 1a).

### 4.2. Hydraulic Measurements

*F. ornus* and *O. europaea* branches were collected in the late afternoon and rehydrated overnight while covered with a black plastic bag after cutting the basal 5 cm underwater. In the early morning, full hydration was checked to ensure that xylem water potential (Ψ_xyl_) was above −0.3 MPa. A group of hydrated branches was used for hydraulic measurements to check possible residual embolism (hydrated group, H, *n* = 5). The remaining branches were dehydrated on the bench, covering several leaves with cling film and aluminum foil to stop transpiration and favor equilibration between leaf and stem xylem in order to measure Ψ_xyl_ with a pressure chamber (mod. 1505D, PMS Instrument Co., Albany, OR, USA). For olive, before bench dehydration, about 15 cm of the 2-year-old stem portions selected for hydraulic measurements and soaking treatment were deprived of leaf blades by cutting them at the insertion point at the petiole and sealing the cut section with impermeable glue (Super Attack, Loctite). Branches were dehydrated until reaching the target Ψ_xyl_, fixed to -(3.5–3.9) MPa for *F. ornus* and to -(4.2–4.7) MPa for *O. Europaea,* based on preliminary assessments. At that point, a group of branches was immediately processed for hydraulic conductivity measurements (dehydration group, D, *n* = 5–6), while a third group was subjected to the soaking treatment as follows. Stem segments, obtained as described below, were sealed at the cut ends with wax tape (Parafilm^®^ M), kept under water and then immersed in a white rectangular bowl containing distilled water, lying horizontally 1 cm below the water level. Stems were rotated by 180° every hour to allow whole stem light exposure. A LED panel (red/blue 96/24) was mounted above the bowl to produce a photosynthetic photon flux density (PPFD) at the water level of about 400 μmol m^−2^ s^−1^. According to Liu et al. [13], most of the embolized vessels of *S. matsudana* refilled already 2 h after branch segment immersion in light conditions. Therefore, duration of soaking was fixed to 2 h for both species (S_2h_ group, *n* = 6). For *F. ornus,* a second set of branches was immersed for 4 h to check if longer rehydration times were needed for hydraulic recovery (S_4h_ group, *n* = 7). After soaking, branch segments were prepared for hydraulic measurements as described below, while a 3 cm adjacent stem segment was taken for Ψ_xyl_ measurements, performed with a dew point hygrometer (WP4-C, Meter Group, Inc., Pullman, WA, USA). To this aim, the segment was quickly wiped with paper towel, cut longitudinally in half, placed in a sample holder and measured upon Ψ_xyl_ stabilization (reached in 30 min–40 min).

Measurements were performed on 3 cm–4 cm long segments of H, D, S_2h_ and S_4h_ stems. For all samples, the base of the branch was trimmed under clean tap water at a distance higher than the maximum vessel length. Maximum vessel length, determined with the “air method” described by Wang et al. [36], averaged 29 cm and 45 cm in *F. ornus* and *O. europaea* branches, respectively. Then, a selected ~15 cm long segment was cut and kept under water. S_2h_ and S_4h_ segments were sealed at both ends with Parafilm and soaked as described above. For hydraulic conductivity measurements, a shorter segment was obtained; the bark was removed from both cut ends, and several thin sharp slides were made with a razorblade at both ends to obtain a 3 cm–4 cm long segment; and the basal end was inserted in a hydraulic apparatus (see [25]).

Xylem hydraulic conductance was measured gravimetrically under a water head of 3.5 kPa, perfusing stems with filtered (0.45 µm) and degassed mineral water added with 10 mM KCl [37]. Hydraulic conductance was measured before (initial hydraulic conductance, *k*_i_) and after (maximum hydraulic conductance, *k*_max_), flushing the sample at high pressure (0.15 MPa) for 3 min to remove xylem embolism. Xylem hydraulic conductivity (*K*) was calculated as

*K* = *k* × L/A (1)

where L is the length of the segment, and A is the sapwood area, calculated as the average sapwood areas measured at the cut ends of the sample.

Percentage loss of hydraulic conductivity (PLC) was then calculated as

PLC = 100 × [1 − (*k*_i_
*k*_max_^−1^)] (2)

### 4.3. Micro-CT Scans and Image Processing

Micro-CT scans were performed at the SYRMEP beamline of the Elettra Synchrotron light source (Trieste, Italy).

To check the capability of *F. ornus* to recover xylem embolism after soil rewetting, four 3-year-old saplings were dehydrated in pots to reach the target Ψ_xyl_ (−3.5 MPa) on 17–21 July 2020. The 2-year-old stem portion of two plants was scanned right after reaching the target Ψ_xyl_ (D_pot_ plants), while the other two were scanned 24 h after re-irrigation to soil field capacity (recovery, R_pot_ plants). Two additional well-watered plants were measured as controls (C_pot_). In all plants, Ψ_xyl_ was measured prior to scanning.

The soaking experiment was performed on potted *F. ornus* saplings on 1 August 2021. All plants were dehydrated by withholding irrigation for about 5 days. Three of them were scanned to detect the embolism level at the target Ψ_xyl_ (D plants). These plants were also used to tests the sample preparation effect on embolism formation, scanning the stem at the same point in three different steps: (i) the intact plant, (ii) the plant cut underwater right above the root collar after immersing the pot (sealed in a plastic bag) in water and (iii) the final stem segment obtained by cutting the stem 14 cm–21 cm above the previous cut, keeping the sample underwater. The length of the segment depended on the length of the 2-year-old stem segment portion, while the scan was always 8 cm–9 cm above the basal cut. After every cut, the cut surface was tightly sealed underwater with Parafilm. An additional scan was performed after cutting the stem segment a few mm above the scanned region in order to observe the fully embolized xylem.

The remaining three plants were prepared in the same way as the D plants, but they were only scanned after soaking the stem segment (obtained at step iii) in water for 2 h (S_2h_ samples) as described above for branches measured with the hydraulic apparatus. This was carried out to avoid the possibility of multiple scans inhibiting cellular activity and possible related processes involved in water uptake and hydraulic recovery [38].

Before micro-CT scanning, all samples were quickly wrapped in cling film and Parafilm to avoid water loss and fixed to the sample holder. The CT studies were performed in propagation-based phase contrast modality using an Orca Flash 4.0 SCMOS, coupled with a 17 µm GGG scintillator, as a detector. The sample was placed 15 cm from the detector, and the pixel size was set at 2.1 µm. The experiment was performed in white beam mode, and 1.0 mm of silica was applied, resulting in a mean X-ray energy of about 22 keV. For each scan, 1800 projections were acquired during the sample rotation over 180°.

The slice reconstructions were performed using SYRMEP Tomo Project (STP) software [39]. A phase retrieval pre-processing algorithm [40] was applied prior to the conventional filtered back-projection algorithm to increase the image contrast.

Reconstructed images were processed using ImageJ (https://imagej.nih.gov/ij/, accessed on 30 November 2021). Due to the larger diameter of the stems compared to the field of view (ca. 4 mm × 4 mm), analyses were conducted on about one-quarter of the stem section, excluding the immature xylem next to the vascular cambium (see Figure 3, Figure 4, Figure S1 and Figure S2). The embolized sapwood area (A_embol_) was calculated by dividing the embolized pixel area by the analyzed sapwood area, expressed as percentage. The embolized vessel area (EVA) was calculated by dividing A_embol_ by the A_embol_ after the final cut above the scanned region (representing the percentage of sapwood area occupied by conduits), expressed as percentage.

### 4.4. Statistics

Statistical analyses were carried out with R (R Core Team, 2017). Boxplot panels were obtained with the “ggplot2” package in R. Bar charts were prepared with SigmaPlot (v. 12.0, Systat Software Inc., Berkshire, UK). For PLC and Ψ_xyl_, the one-way ANOVA test (response variable ~f(treatment)) through the *aov* function was applied, followed by Tukey’s HSD post hoc test (only for significant ANOVA, *p* < 0.05) through the *TukeyHSD* function in the “stats” package after checking for normality of residuals and homogeneity of variances. When homogeneity of variance assumption was violated, generalized least squares (GLS) models were calculated with the *gls* function, including a “varIdent” variance structure, in the “nlme” R package [41], followed by Tukey’s HSD post hoc analysis (for significant tests), with *p*-values adjusted using the Bonferroni–Holm method. The effect of progressive cuttings on A_embol_ and EVA in the micro-CT test experiment was tested using linear mixed models (LMMs) through the *lme* function. Specifically, one LMM was fitted by separately setting A_embol_ or EVA as the response variable and by setting the cutting stage as the explanatory one, with the plant replicate as the random effect. Pairwise comparisons were performed through *lsmeans* function in R package “emmeans” [42].

## 5. Conclusions

In this work, we tested the hypothesis that the immersion of photosynthesizing stem segments in distilled water for some hours in the presence of light would induce hydraulic recovery through bark water uptake in the embolized branches of two drought-resistant Mediterranean tree species (*F. ornus* and *O. europaea*). Hydraulic recovery was previously reported for the soaked stem segments of *S. matsudana*, and it was related to the generation of an osmotic gradient [13]. However, for the two species analyzed in this study, which are known to be capable of hydraulic repair under moderate tension (additionally demonstrated here by an in vivo micro-CT analysis of the potted saplings of *F. ornus*), refilling by bark water uptake did not occur. Our data suggest that refilling does not commonly occur in cut stems soaked under water, thus suggesting that this common procedure in hydraulic measurements is not likely to produce artefactual results. Clearly, further soaking studies under light conditions should be performed on photosynthesizing stems of other species to better understand both the potential and the limits of this process, as well as the possible consequences in terms of the accuracy of hydraulic measurements of xylem embolism.

## Data Availability

The data presented in this study are available in the Supplementary Materials.

## Supplementary Materials

The following are available online at www.mdpi.com/article/10.3390/plants11030307/s1, Figure S1: X-ray micro-CT transverse images of stems of drought-stressed 2-year-old *F. ornus* saplings, Figure S2: Cutting effect on embolism formation in *F. ornus* potted saplings visualized with micro-CT. Figure S3: X-ray micro-CT transverse images of 2-year-old *F. ornus* stem segments after the soaking treatment (S_2h_).
