# Supplementary material for: No Evidence for Light-Induced Embolism Repair in Cut Stems of Drought-Resistant Mediterranean Species under Soaking
Source: Plants (Basel). 2022 Jan 24;11(3):307. doi: 10.3390/plants11030307 (PMC8840644; doi:10.3390/plants11030307)
Supplement: Supplementary file 1 [file plants-11-00307-s001.zip › plants-1540587-supplementary.pdf]

plants

Supplementary material

Article title: No evidence for light-induced embolism repair in cut stems of drought-resistant Mediterranean species under soaking

Martina Tomasella[1], Sara Natale[1], Francesco Petruzzellis[1,2], Sara Di Bert[1], Lorenzo D'Amico[3,4], Giuliana Tromba[3] and Andrea Nardini[1,*]

[1] Dipartimento di Scienze della Vita, Università di Trieste. Via L. Giorgieri 10, 34127 Trieste (Italy);
[2] Dipartimento di Scienze Agroalimentari, Ambientali e Animali, Università di Udine. Via delle Scienze 91, 33100 Udine, Italy;
[3] Elettra-Sincrotrone Trieste, Area Science Park, 34149, Basovizza, Italy
[4] Dipartimento di Fisica, Università di Trieste. Via A. Valerio 2, 34127 Trieste (Italy)
* Correspondence: nardini@units.it.

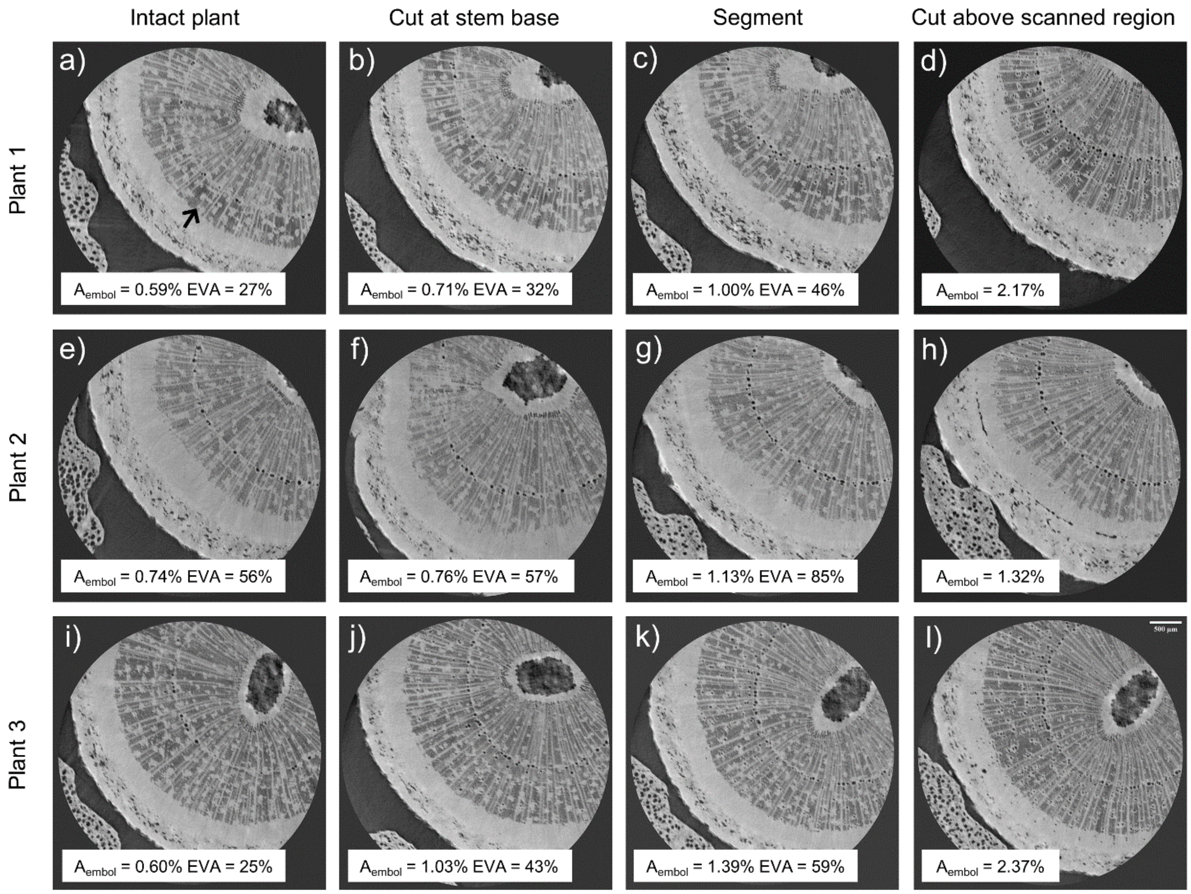

Figure S1. X-ray Micro-CT transverse images of drought stressed 2-year-old *F. ornus* saplings (n=3). Scans were performed in drought-stressed intact plants (a,e,i), after subsequent cut underwater at the base of the stem (b,f,j), after a second cut underwater to obtain a stem segment (c,g,k) and after cutting above the scanned region to embolize all mature conduits (d,h,l). A_{embol} = percentage of embolized sapwood area; EVA = percentage of embolized vessell area, all calculated excluding the immature sapwood close to the cambium (see arrow delimiting mature-immature sapwood in a).

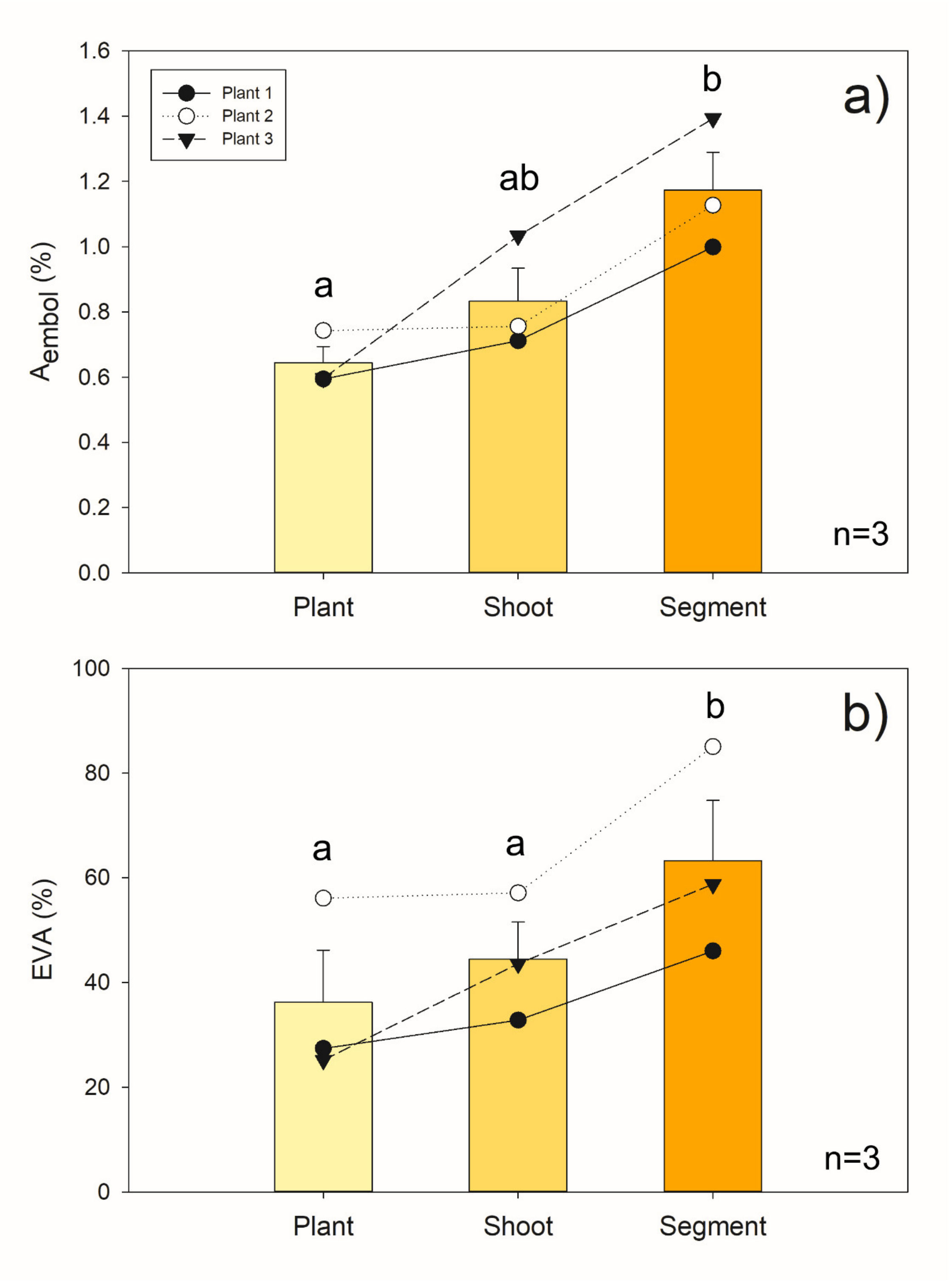

Figure S2. Cutting effect on embolism formation in *F. ornus* potted saplings visualized with Micro-CT. **a**) Percentage of embolized sapwood area (A_{embol}) and **b)** percentage of embolized vessel area (EVA) measured in Micro-CT transverse images of drought-stressed saplings. Plants (1-3, indicated by symbols, n=3) were consecutively scanned when intact (Plant), after subsequent cut underwater at the base of the stem (Shoot) and after a second cut underwater to obtain a stem segment (Segment). Values are means ± SE. Different letters denote statistically significant differences among Plant, Shoot and Segment ($P < 0.05$).

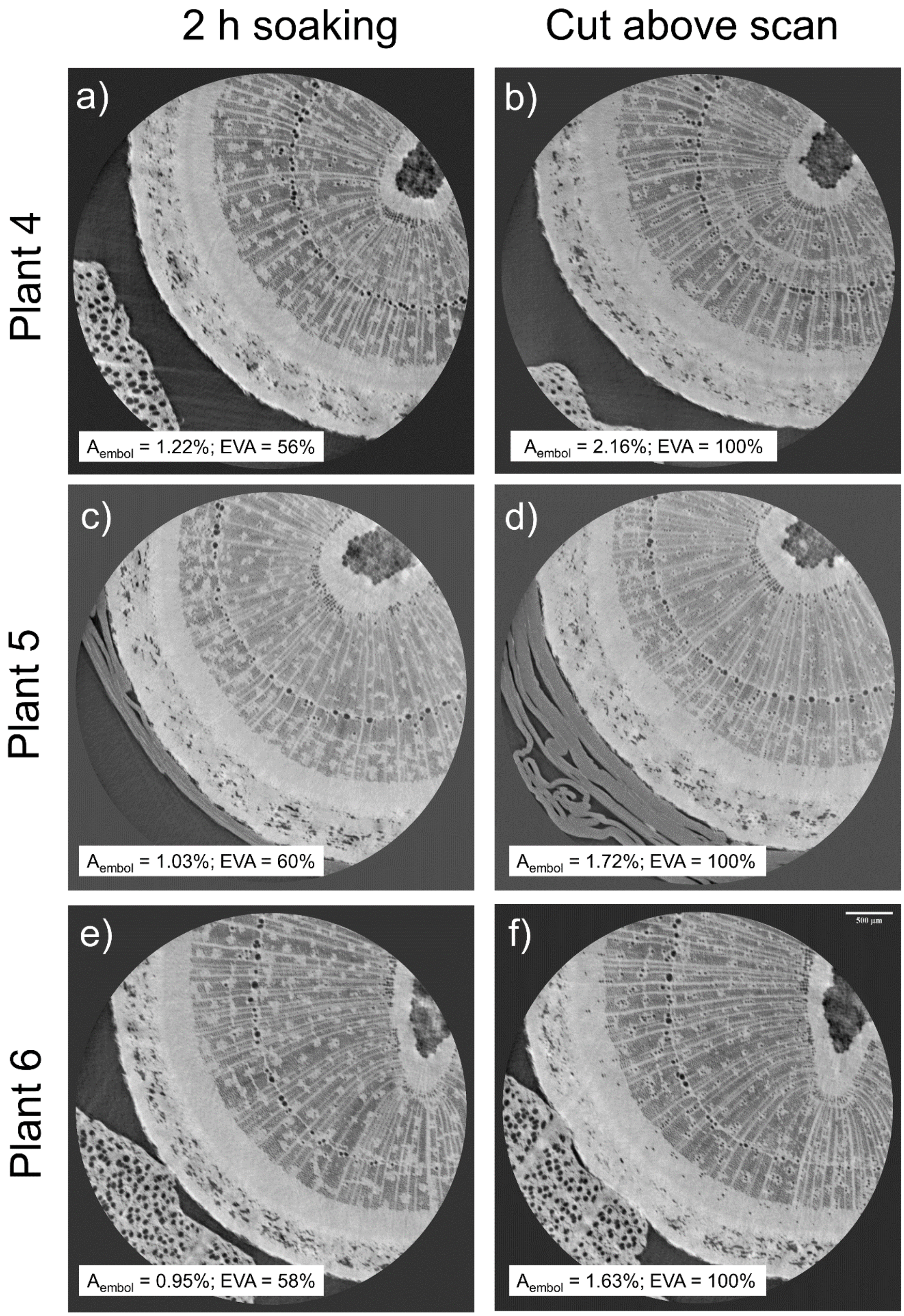

Figure S3. X-ray Micro-CT transverse images of 2-year-old *F. ornus* stem segments after the soaking treatment (S_{2h}, n=3). Scans were performed in drought-stressed segments soaked under light for 2

hours (a,c,e) and after cutting above the scanned region to embolize all mature conduits (b,d,f). A_{embol} = percentage of embolized sapwood area; EVA = percentage of embolized vessell area.
